# Assessment of classification accuracy in endodontics by dental students: a comparative study of holographic imaging and conventional radiography

**DOI:** 10.1186/s12909-026-10035-x

**Published:** 2026-07-29

**Authors:** Maximilian Louis Lederer, Anna Greta Barbe, Malin Janson, Max von Kohout, Christoph Matthias Schoppmeier

**Affiliations:** 1 Polyclinic for Operative Dentistry and Periodontology, University of Cologne, Faculty of Medicine and University Hospital Cologne, Cologne, Germany; 2 Department of Prosthetic Dentistry, University of Cologne, Faculty of Medicine and University Hospital Cologne, Cologne, Germany; 3 Institute of Medical Statistics and Computational Biology, University of Cologne, Faculty of Medicine and University Hospital, Cologne, Cologne, Germany

**Keywords:** Education, Dental, Endodontics, Holography, Radiography, Three-dimensional imaging

## Abstract

**Background:**

Dental students must master endodontic anatomy, radiographic diagnostics, and treatment strategies early in their clinical training—yet traditional teaching methods, including two-dimensional radiographs, often fail to effectively convey complex spatial structures. Hologram technology has emerged as a promising tool for three-dimensional visualization in dental education. The aim of the study was to compare the classification accuracy of dental students using holograms versus conventional periapical radiographs in identifying endodontic structures.

**Methods:**

This randomized single center-controlled trial included 79 dental students (years 4–6). After a calibration seminar and pre-test each of the 79 students assessed six tooth cases—three presented as holograms and three as periapical radiographs. Holograms were created from cone-beam computed tomography data, annotated by two calibrated examiners to establish reference points regarding the number of canals, Vertucci classification, and treatment complexity. Students evaluated each case for these parameters; responses were compared to the reference points to assess classification accuracy. The final evaluation form captured their subjective perceptions of the new hologram system. Primary binary outcomes were analyzed using logistic mixed-effects models. *P*-values were adjusted for multiple testing using the Benjamini–Hochberg procedure. Statistical significance was set at *p* < 0.05.

**Results:**

After Benjamini–Hochberg correction, holography significantly improved root-canal number identification in three multi-rooted cases (adjusted *p* ≤ 0.007). For Vertucci classification, only the maxillary anterior tooth remained significant (adjusted *p* = 0.044). Treatment-complexity assessment showed case-dependent results, favoring radiography in two cases (adjusted *p* = 0.045 and *p* = 0.006) and holography in one case (adjusted p = 0.013).

**Conclusions:**

Holographic visualization demonstrated case- and task-specific effects in endodontic training. Holograms were able to improve classification accuracy in certain endodontic tasks. However, they did not outperform conventional periapical radiographs in all outcomes. Holography could be an effective, supplementary tool for teaching certain endodontic tasks, but its application requires further investigation.

**Trial registration:**

The study was prospectively registered in the German Clinical Trials Register (DRKS-ID: DRKS00035000) on 13.09.2024.

**Supplementary Information:**

The online version contains supplementary material available at 10.1186/s12909-026-10035-x.

## Background

Periapical lesions, persistent symptoms, and long-term failure of endodontic treatments are frequently linked to undetected or insufficiently treated root canals—largely due to the considerable anatomical complexity and variability of root canal systems [1–3]. A precise understanding of root canal morphology is therefore essential for clinical success in endodontics.

Missed canals are among the leading causes of endodontic failure, often resulting in persistent periapical pathology, ongoing symptoms, or the need for retreatment [4]. A basic understanding of the anatomy of the root canal system is essential for successful endodontic treatment. However, diagnostic inaccuracies and errors can arise from gaps in theoretical knowledge and the complex challenge of interpreting three-dimensional root canals on two-dimensional radiographs. Undergraduate dental students are increasingly reporting difficulties in understanding root canal anatomy and interpreting X-rays. In addition, students’ confidence in their own endodontic skills appears to grow with increasing clinical experience, although treating more complex cases, such as molars, remains a particular challenge [5, 6].

Traditional instructional approaches rely heavily on two-dimensional visual resources, such as schematic illustrations, cross-sectional anatomical diagrams, and intraoral radiographs [7, 8]. While these tools remain fundamental in teaching root canal morphology, they are inherently limited in conveying the three-dimensional complexity and structural variability of root canal systems [9]. As a result of this inadequate representation, students may have difficulty developing a three-dimensional understanding of root canal anatomy, which, given the complexity of the root canal system, could increase the risk of diagnostic oversights in clinical practice [10].

This challenge is further exacerbated by the significant anatomical variations observed in posterior teeth. In particular, molars often exhibit multiple principal canals, abrupt curvatures, merging pathways, and complex apical or lateral ramifications [3, 11]. Additional variations, such as C-shaped canals in mandibular second molars, isthmus-like connections, taurodontism, or accessory roots, may further complicate the radiographic interpretation of root canal anatomy, particularly for novice clinicans.

To address these challenges, advanced imaging modalities such as cone-beam computed tomography (CBCT) have increasingly been adopted, demonstrating notable success in improving anatomical visualization. Although CBCT is part of postgraduate training, interpreting complex root canal anatomies—especially atypical or rare configurations—remains cognitively demanding for less experienced learners and is typically more suitable for postgraduate-level training [12, 13]. In addition to traditional teaching methods and three-dimensional radiographs, students today have access to more digital resources, such as web-based learning platforms and online videos of three-dimensional anatomical structures [14]. Currently, however, these resources are not standard in dental education and are primarily used for self-study.

In response to these limitations, contemporary dental education must go beyond the adoption of diagnostic imaging and incorporate didactic technologies that actively promote three-dimensional anatomical comprehension. Immersive media such as virtual reality (VR) and augmented reality (AR) have shown promising results in early medical education by enabling interactive engagement with complex anatomical structures and supporting the transition from two-dimensional perception to three-dimensional mental modeling [8, 15]. Recent digital and immersive approaches to improving endodontic education have already been shown to have a positive impact on student motivation, engagement, and learning outcomes [16].

Expanding on this immersive potential, holographic visualization represents a particularly forward-looking and underutilized didactic approach [17]. Unlike screen-based simulations or headset-dependent systems, holograms project three-dimensional anatomical data directly into real space, visible without auxiliary devices such as glasses or VR gear [18, 19]. By providing students with an intuitive visualization of complex anatomical structures from multiple perspectives, holograms could enhance spatial understanding and offer a new method for interactive and collaborative learning [20]. Although holographic visualization has already been demonstrated as a promising method, its targeted use in dental education, especially in endodontics, remains insufficiently investigated [19, 21].

It has thus already been demonstrated that immersive technologies such as virtual or augmented reality and digital learning methods can improve students’ understanding of root canal anatomy [8, 16]. Furthermore, it is technically possible to display endodontic anatomy holographically [13]. Nevertheless, the evidence to date remains incomplete regarding the use of free-floating holographic projection systems in endodontic education. In particular, the objective accuracy of students in performing endodontic tasks, such as classifying the number of root canals, canal configuration, and treatment complexity, has not yet been sufficiently investigated.

We therefore comprehensively evaluated the diagnostic and didactic potential of holographic visualization compared to conventional radiographic imaging within undergraduate endodontic training. In particular, we evaluated the classification accuracy of dental students in determining canal number, classifying canal configurations according to Vertucci’s system, and anticipating treatment complexity. In addition, subjective parameters such as usability, perceived educational value, and the innovation potential of holographic technology were comprehensively evaluated. Our findings were intended to support the evidence-based development of seminar-based teaching formats and promote the integration of immersive visualization into endodontic education—with the overarching goal of enhancing the competence of future practitioners in three-dimensional diagnosis.

To the best of our knowledge, our study is the first randomized study to evaluate the use of free-floating holographic projections for classifying the number of root canals, canal configuration, and treatment complexity, and to compare their classification accuracy with that of conventional periapical radiographs.

Based on the idea that immersive three-dimensional visualization can improve understanding of anatomical structures, we hypothesize that holographic visualizations are superior to conventional periapical radiographs in terms of both objective classification accuracy and subjective perception of teaching effectiveness. Because this investigation was exploratory, the findings were interpreted with an emphasis on case- and task-specific effects.

## Methods

### Study setting and participants

Our single center randomized controlled trial was open to all available dental students enrolled in four interdisciplinary clinical courses 1–4 (IC 1–4) during the fall semester of 2024/2025. These courses involve independent patient treatment and take place between the seventh and tenth semesters of study. Consequently, the students have varying levels of practical experience and theoretical knowledge. Inclusion criteria were age ≥ 18 years, active enrollment in one of the four endodontic courses, and provision of written informed consent. Exclusion criteria comprised neurological conditions (for example epilepsy, migraine), visual intolerance to holographic projection, and absence of informed consent. To ensure confidentiality, all student data were pseudonymized prior to analysis.

**Fig. 1:**
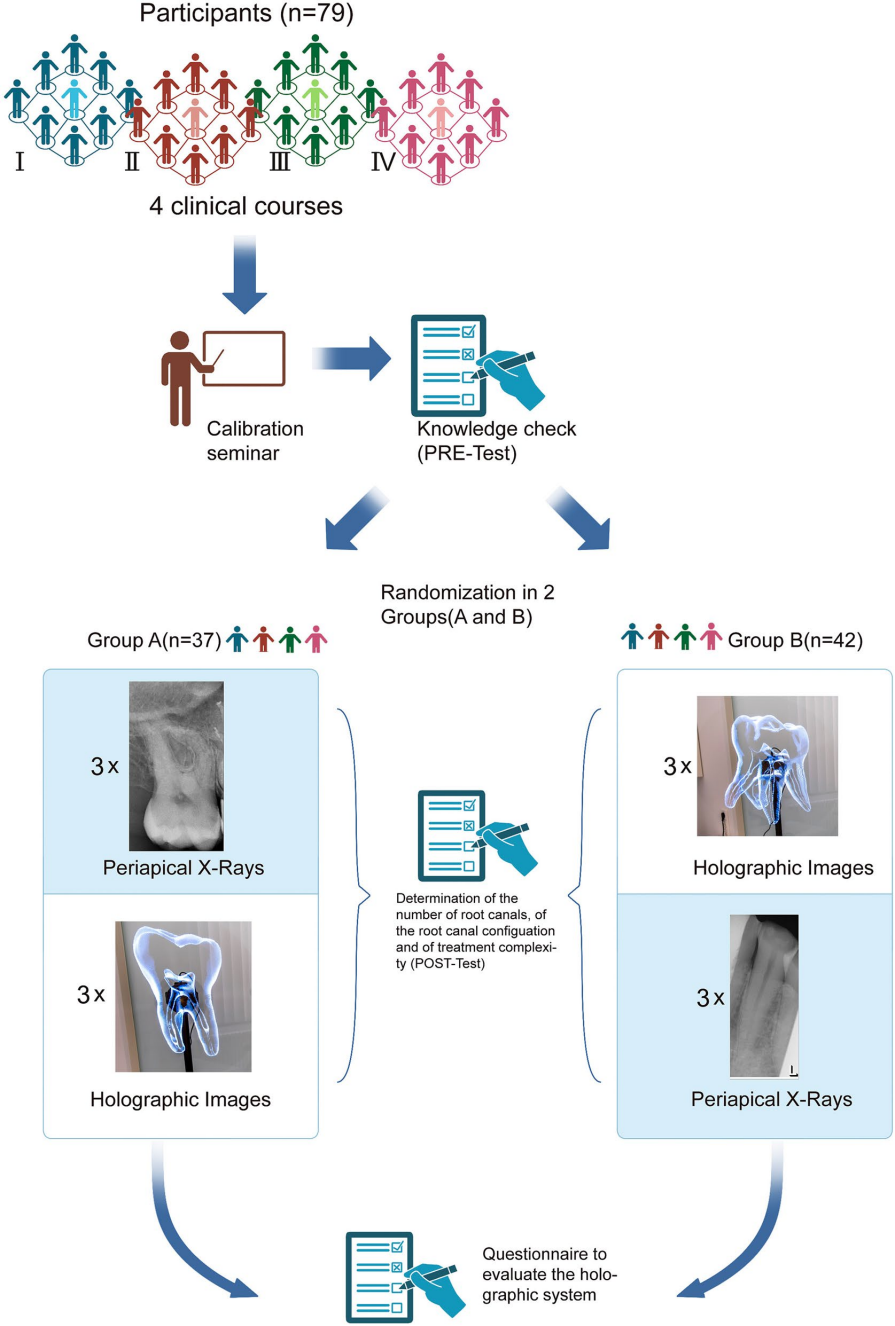
Study design and procedures

Ethical approval was granted by the appropriate Ethics Committee of the Medical Faculty Cologne (Approval No. 24–1196) and the study was prospectively registered in the national clinical trials register (DRKS-ID: DRKS00035000, Date of registration: 13.09.2024). The study was conducted in accordance with the CONSORT 2025 guidelines. All participating students provided written informed consent for study participation and data processing prior to enrollment. An overview of the study design is presented in Fig. 1.

### Interventions

Cone-beam computed tomography data sets acquired between March 2023 and March 2024 as part of general medical care within the department for Oral and Maxillofacial Surgery at the University Hospital were retrospectively reviewed by the first author. CBCT scans are used because they are the most precise radiographs used in clinical treatment and are regularly employed in endodontic diagnostics. Only cases in which pulp structures and root canal anatomy were clearly identifiable, accompanied by corresponding periapical radiographs, were included in the study. To minimize radiation and image artifacts that could complicate radiological assessment, teeth with coronal restorations, root fillings, or other radiopaque materials were excluded. The CBCT datasets and periapical radiographs used were not aquired on the same date. The maximum time interval between the radiographs was four months. To ensure comparability between the images, only case depicting the same anatomical conditions were included. Cases were excluded if there was any indication for changes due to dental interventions or pathologies that could affect the anatomical conditions. A total of six teeth, each representing a major tooth group (maxillary and mandibular anterior teeth, premolars, and molars), were selected to represent all major tooth groups and all three levels of treatment complexity as defined by the AAE [22].

Two experienced endodontists independently classified all six cases with respect to root-canal number, Vertucci configuration and treatment complexity. The two evaluators are certified endodontists with formal training in CBCT interpretation. They both received an overview of the radiologically assessable criteria and their corresponding classification within the three treatment complexity categories defined by the AAE. The reference classification regarding treatment complexity established by the two expert examiners was based on the same radiographic criteria available to the students to ensure comparability between reference and participant assessments. Ultimately, three cases were annotated as having low treatment complexity, two as having moderate treatment complexity, and one as having severe treatment complexity. Interrater agreement was assessed using Cohen´s kappa statistics (linearly weighted kappa for the ordinal outcomes root-canal number and treatment complexity). In instances of disagreement, consensus was reached to establish the reference points.

#### Vertucci classification

The Vertucci classification has gained widespread acceptance as an international standard to systematically capture the extensive morphological diversity of root canal systems, both in clinical practice and dental education [23]. This classification provides standardized terminology for diagnostics, treatment planning, and scientific communication, while also serving as a pedagogical foundation for the structured categorization of anatomical variants.

The complexity and difficulty of endodontic treatment are influenced by numerous factors [22]. In addition to patient- and treatment-specific factors, radiological findings such as the course of the canal also play an important role in this classification. According to these factors determined by the American Association of Endodontists (AAE), endodontic cases can be classified into three treatment complexities, which may necessitate adjustments to the time frame and the required materials, as well as referrals to more specialized colleagues.

#### Preparation of three-dimensional models

**Fig. 2:**
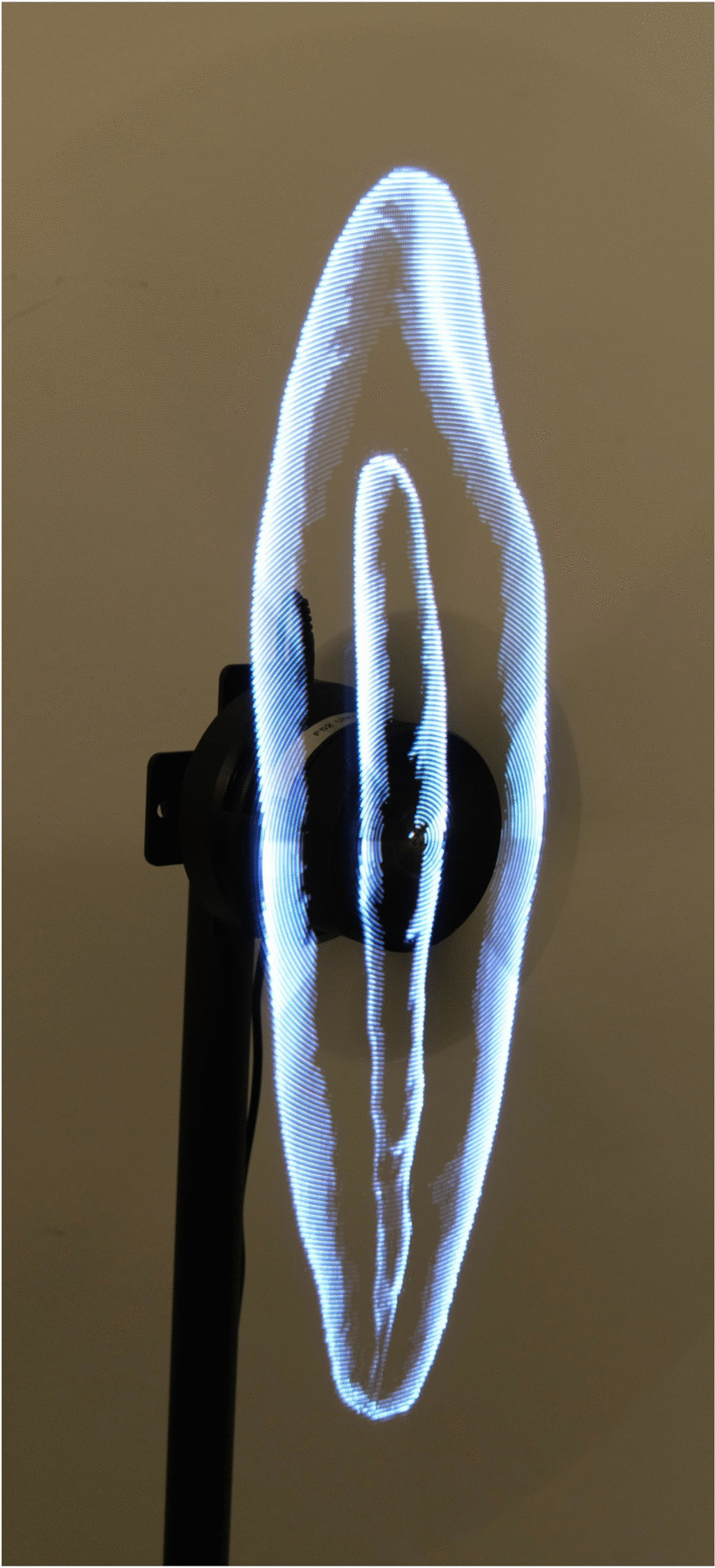
Holography lower anterior teeth

**Fig. 3:**
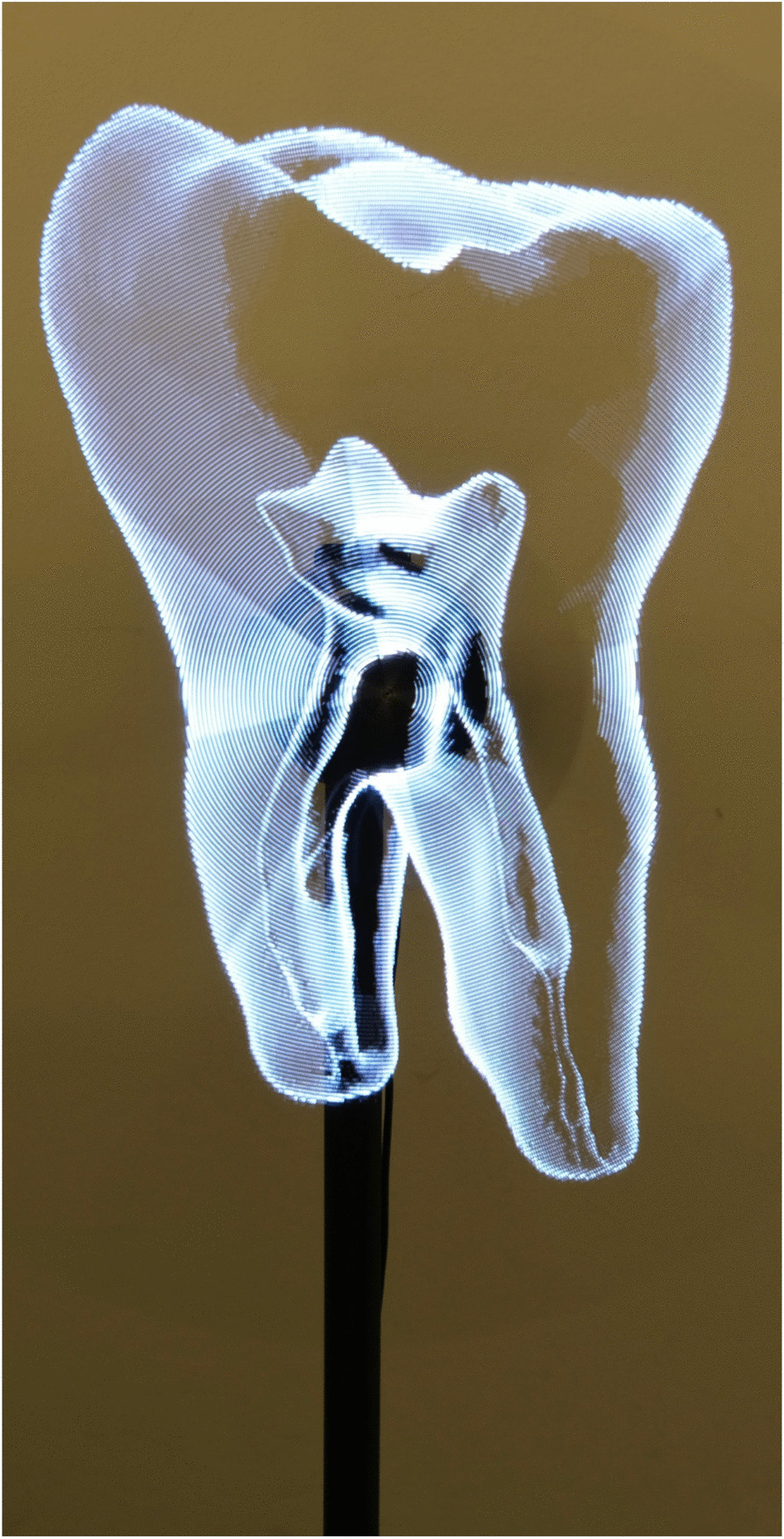
Holography mandibular molar

**Fig. 4:**
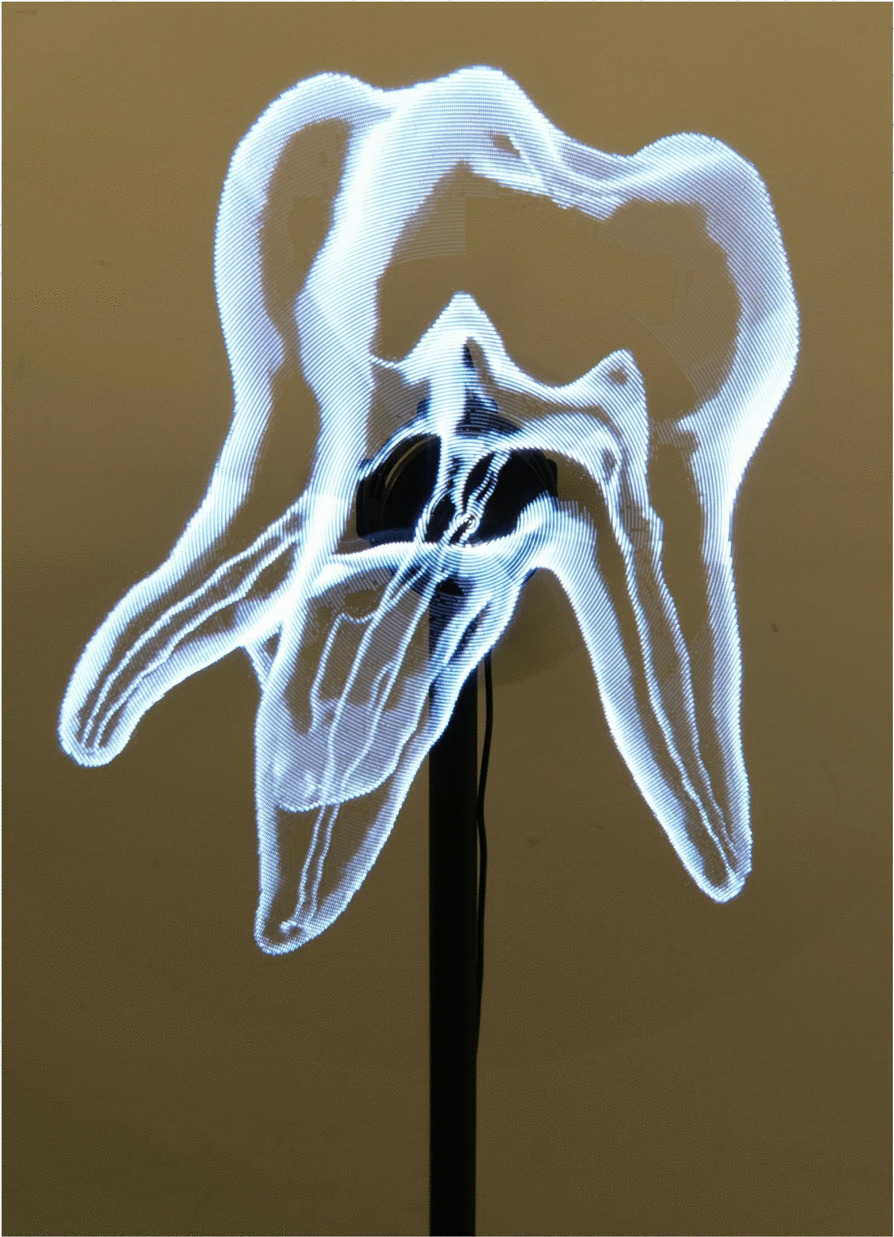
Holography maxillary molar

The selected CBCT datasets were segmented using the open-source software 3D Slicer (Version 5.4.0, 2023) [24] and converted into STL files. The resulting digital 3D models were refined in Blender (Foundation SB. Community BO, Amsterdam, 2018) and prepared for 360° animation. Final video rendering was performed using Adobe Premiere (Systems A. Adobe Inc. Adobe Premiere Pro, version 24.1 San Jose (CA), 2024) and exported to the SpinDisplay application (房 小. SpinDisplay, 2021). Holographic visualization was achieved via a volumetric 3D projector (Dongguan Fankai Hardware Products Co., Guangdong), enabling free-floating, three-dimensional representation without the need for headsets or special glasses. The hologram projector was mounted on a 120-cm-high tripod. The diameter of the holographic projection is 52 cm. The holograms for the six cases were designed as an animation rendered in advance in Blender, which included a 360° rotation around the vertical tooth axis and a zoom in on the pulp structures. The animation was 1 min and 20 s in duration per case, and each case was shown to the students twice. The total duration of the presentation was thus 2 min and 40 s. The animation had a resolution of 1920 × 1080 pixels and ran at a rate of 25 frames per second. All data processing was conducted on secure institutional workstations within the university network. To illustrate the holograms, selected cases are exemplarily shown in Figs. 2, 3 and 4.

#### Calibration seminar and pre-test

Students received a 45-min lecture to provide an overview of root canal anatomy, the Vertucci classification system, and the radiologically assessable criteria of the AAE Case Difficulty Assessment Form. Emphasis was placed on radiological criteria such as canal configuration, canal curvatures, and canal visibility. No patient- or treatment-specific criteria were covered.All seminar sessions were conducted by the same experienced clinician to ensure consistent instructional quality. Participation was open to students not enrolled in the study. To address any differences in the participants’ prior knowledge, a pre-test was administered following the calibration seminar. The pre-test was not intended to assess the actual educational impact of the intervention, but rather to gauge participants’ individual prior knowledge regarding root canal anatomy, the Vertucci classification, and the AAE’s complexity classification. The 15-min single-choice pre-test comprises a series of 20 questions concerning the conventional tooth group-specific quantity of root canals, the eight distinct Vertucci configurations, and the classification of cases into one of the three treatment complexities as delineated by the AAE. The pre-test was scored as a percentage of the maximum possible score (100%). Questions 1–6 assessed knowledge of the number of root canals typically found in the six major tooth groups. A correct answer was awarded 5%, an answer deviating by one canal was awarded 2.5%, and an answer deviating by two or more canals received 0%. The remaining 14 questions addressed the Vertucci classification and the AAE treatment complexity criteria. These questions were scored dichotomously, with 5% awarded for a correct answer and 0% for an incorrect answer.

#### Post-test

After the pre-test, each participant assessed six clinical cases (maxillary/mandibular anterior teeth, premolars, molars), with three teeth presented as radiographs and three as holograms, depending on group allocation. The distribution of tooth groups among the six cases is documented below: the first case involved a maxillary molar, the second involved a mandibular premolar, the third involved a maxillary anterior tooth, the fourth involved a mandibular anterior tooth, the fifth involved a maxillary premolar and the sixth case involved a mandibular molar. Participants answered three questions about each of the six cases: (1) the number of root canals, (2) the Vertucci classification, and (3) the treatment complexity according to the AAE criteria. The score for each case was expressed as a percentage of the maximum possible score (100%). For the assessment of root canal number, a correct answer received 33% of the maximum score, an answer that differed by one canal received 17%, and an answer that differed by two or more canals received 0%. The Vertucci classification and treatment complexity were scored dichotomously: 33% for a correct answer and 0% for an incorrect answer. The resulting scores were incorporated into statistical models to estimate the probability of correctly identifying the findings for each visualization modality.

In group A, cases 1 to 3 were presented using conventional periapical radiographs, while cases 4 to 6 were presented using hologram technology. In group B, this presentation order was reversed. To avoid recall effects that would have occurred if participants assessed the same case twice using different visualization modalities, a crossover design with two study groups was chosen. While the assignment of visualization modalities and the allocation of cases to each visualization method were randomized, the chronological order in which the cases were assessed remained the same in both groups.

Assessments were conducted under standardized conditions in a clinical treatment room. Students were allotted 3 min per case, yielding a total assessment time of 18 min. The students were seated at approximately one meter from the holographic projection. The treatment room was dimmed by closed roller blinds, and the lights were extinguished to ensure optimal visibility of the holograms. All radiological features considered for the complexity assessment, including canal configuration, canal curvatures, and canal visibility were represented in all images. The holograms were generated directly from the CBCT data sets and thus depicted the same anatomical characteristics. These criteria were also visible in the periapical radiographs, although the limitations of two-dimensional images must be considered.

Following the diagnostic phase, students completed a questionnaire evaluating usability, perceived educational value, and suggestions for improvement. The responses to these questions were collected using either a visual analog scale (VAS) ranging from 0 to 10 (equivalent to 0–100%) or a 5-point Likert scale. Since no validated questionnaires exist in this specific area of application, an exploratory questionnaire was developed based on the survey instruments used in comparable studies [25]. This exploratory questionnaire was not validated prior to the start of the study.

### Outcomes

The primary endpoint was the proportion of correct answers from three separately examined categories: number of root canals, Vertucci classification, and treatment complexity depending on the visualization method.

Secondary outcomes consisted of subjective ratings of the hologram technology in terms of usability, perceived innovation potential, and its applicability in dental education.

### Statistical analysis

#### Sample size

The practically feasible sample size of 90 subjects (expected number of students in interdisciplinary courses 1–4 in the winter semester 24/25) is compatible with the current state of research in pilot studies [26–30]. With a fixed significance level for the α error of 5% and an assumed mean effect size of 0.3 (according to Cohen’s d), it is possible to achieve a statistical power of 80% (1–β) with a sample size of 90 subjects. This estimate was calculated using the G-Power program (version 3.1.9.4) based on a paired t-test and considers an expected dropout rate of 10%. Because comparable data is lacking, our case number estimate is based on a pragmatic, exploratory approach.

#### Randomization and allocation

Following the calibration seminar, a person who was not involved in conducting the study randomized participants using block randomization with alternating block sizes of 4 and 6 to ensure a balanced group assignment between groups A and B. This group assignment determined the order of exposure to the holographic and radiographic cases. An individual not involved in the study execution was granted access to the randomization list, thereby ensuring the equitable allocation of groups in the event of any ambiguities. Due to the study design, it was not possible to blind the subjects or investigators.

#### Statistical methods

All statistical analyses were conducted using R statistical software, version 4.3.3. The three primary binary outcomes were analyzed using separate logistic mixed-effects models with the glmer function from the lme4 package. For each outcome, the dependent variable indicated whether the response was correct. The fixed effects included treatment modality (holography = 1; periapical radiography = 0), case as a six-level categorical factor, and their interaction. Participant ID was included as a random intercept to account for repeated observations within participants. Standardized pre-test performance was included as a covariate. Semester affiliation was not included in the primary mixed-effects models and was examined separately in exploratory analyses. The models were fitted by maximum likelihood using the BOBYQA optimizer. Estimated marginal probabilities and case-specific contrasts were obtained using the emmeans package. Missing outcome data were not imputed, and all available observations were analyzed under the missing-at-random assumption. Wald-based 95% confidence intervals and *p*-values were calculated. For each of the six cases, we compared holography and periapical radiography separately for the three outcome domains, resulting in 18 case- and outcome-specific contrasts. The *p*-values for these contrasts were adjusted across all 18 comparisons using the Benjamini–Hochberg false discovery rate procedure, and statistical significance was set at an adjusted *p* < 0.05.

Because the visualization modality in the present case-dependent crossover design is inextricably linked to individual cases, the results were interpreted as exploratory, case-specific comparisons rather than as proof of the superiority of one visualization method over another.

## Results

### Interrater reliability reference points

There was high interrater agreement between the two expert examiners for root canal number (5 out of 6 cases, or 83%; Cohen’s kappa = 0.77; linearly weighted kappa = 0.86) and treatment complexity (5 out of 6 cases, or 83%; Cohen’s kappa = 0.67; linearly weighted kappa = 0.67). For the Vertucci classification, there was agreement in four of six cases (67%; Cohen’s kappa = 0.25). Since the Vertucci classification represents nominal categories rather than an ordinal scale, a weighted kappa was not calculated. All disagreements (root canal number: Case 2; treatment complexity: Case 2; Vertucci classification: Cases 2 and 6) were resolved by consensus before the student assessments.

### Participants

**Fig. 5:**
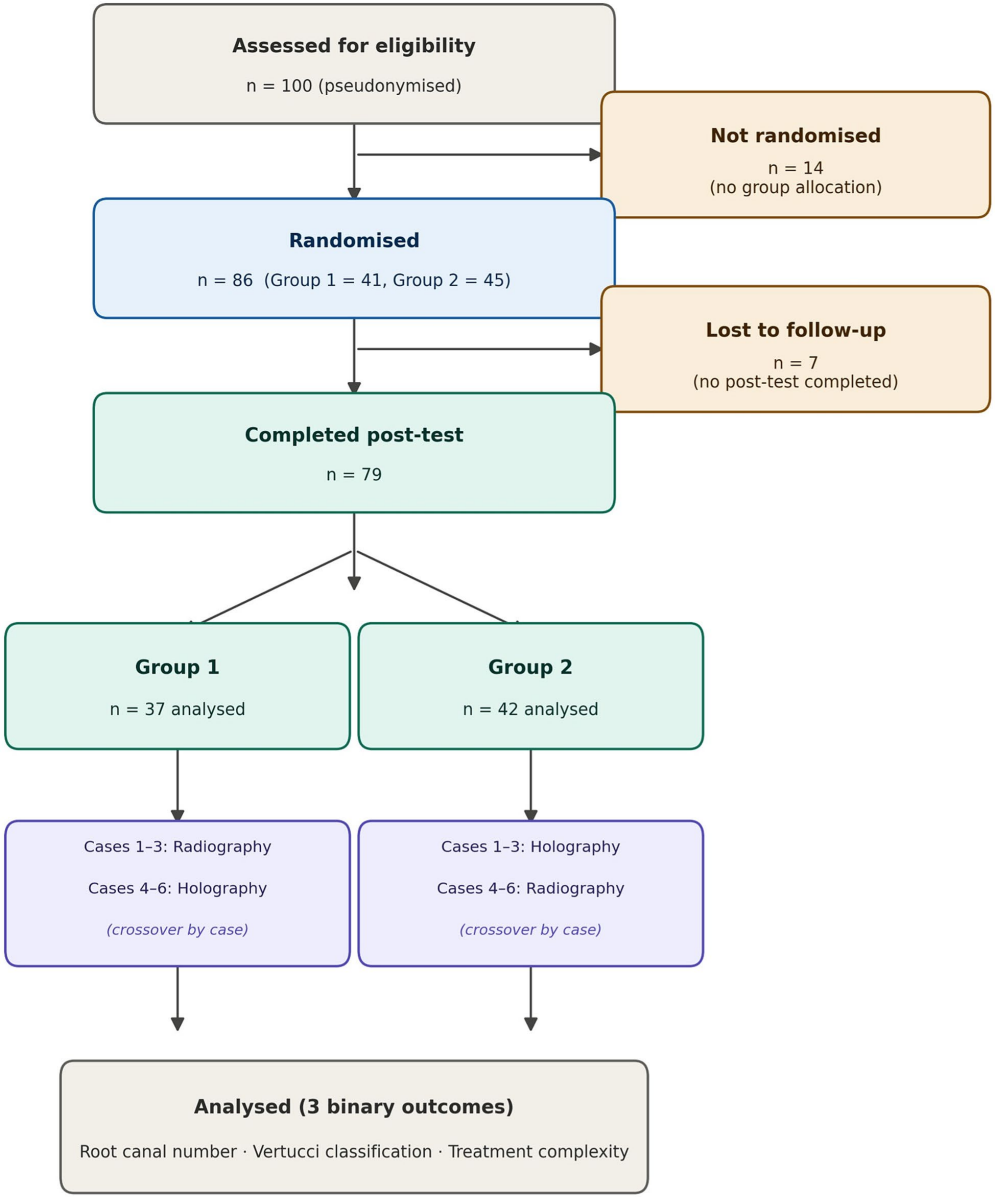
CONSORT flow diagram

Of a total of 100 potential participants in course IC1-4, 86 opted to participate in the study. 79 students completed the post-test and were included in the analysis (Fig. 5). Across all outcomes, only one post-test observation was missing, which was addressed using the mixed-effects model without imputation. The demographic composition of the participants is presented in Table 1.

**Table 1:** Demographic composition of participants

Category	Group A ^†^ (*n* = 37)	Group B ^†^ (*n* = 42)	Total (*N* = 79)
Age (years), mean ± SD (min, max)	—	—	27.0 ± 5.05 (21, 51)
Gender, n (%)			
Male	10 (27.0)	14 (33.3)	24 (30.4)
Female	27 (73.0)	28 (66.7)	55 (69.6)
Group A/B (%)	—	—	46.8/53.2
Course affiliation, n (%)			
IC1	8 (21.6)	8 (19.0)	16 (20.3)
IC2	6 (16.2)	8 (19.0)	14 (17.7)
IC3	14 (37.8)	18 (42.9)	32 (40.5)
IC4	9 (24.3)	8 (19.0)	17 (21.5)

*Abbreviations*: *IC* interdisciplinary course (IC 1–4 correspond to semesters 7–10, respectively), *n* number of participants, *N* total number of participants

^†^Group A = cases 1–3 visualization as periapical radiograph, cases 4–6 visualization as holography; group B = cases 1–3 visualization as holography, cases 4–6 visualization as periapical radiograph

Analysis revealed that no differences between the two groups (A and B) in terms of course enrollment *(p* = 0.912), age of the participants *(p* = 0.933), and gender distribution *(p* = 0.543).

### Pre-test

The mean score attained in the pre-test was 89.5 ± 8.2% (Table 2).

**Table 2:** Performance of the participants in the pre-test

No	Question/Statement	Mean score (%)	SD	Min score (%)	Max score (%)
1	How many canals do maxillary anterior teeth typically have?	4.87	0.790	0.0	5.0
2	How many canals do maxillary premolars typically have?	4.84	0.834	0.0	5.0
3	How many canals do maxillary molars typically have?	4.56	0.961	0.0	5.0
4	How many canals do mandibular molars typically have?	4.75	0.858	0.0	5.0
5	How many canals do mandibular premolars typically have?	4.72	1.058	0.0	5.0
6	How many canals do mandibular anterior teeth typically have?	4.72	0.894	0.0	5.0
7	What kind of channel configuration is Vertucci type 1?	4.75	1.103	0.0	5.0
8	What kind of channel configuration is Vertucci type 2?	3.16	2.425	0.0	5.0
9	What kind of channel configuration is Vertucci type 3?	3.99	2.022	0.0	5.0
10	What kind of channel configuration is Vertucci type 4?	4.05	1.974	0.0	5.0
11	What kind of channel configuration is Vertucci type 5?	3.86	2.111	0.0	5.0
12	What kind of channel configuration is Vertucci type 6?	4.11	1.921	0.0	5.0
13	What kind of channel configuration is Vertucci type 7?	4.05	1.974	0.0	5.0
14	What kind of channel configuration is Vertucci type 8?	4.75	1.103	0.0	5.0
15	How many treatment complexities are there according to the AAE?	4.68	1.225	0.0	5.0
16	How many and which factors are required for a classification in the complexity “low”?	4.94	0.563	0.0	5.0
17	How many and which factors are required for a classification in the complexity “moderate”?	4.75	1.103	0.0	5.0
18	How many and which factors are required for a classification in the complexity “high”?	4.87	0.790	0.0	5.0
19	Which groups of teeth count as a moderate treatment complexity?	4.30	1.742	0.0	5.0
20	Which groups of teeth count as a severe treatment complexity?	4.75	1.103	0.0	5.0
	Total percentage achieved:	89.46	8.172	70	100

Assessment items to generate baseline knowledge. Item-level values are reported as mean scores in percentage points, with a maximum of 5 percentage points per item. The total pre-test score is reported as the mean percentage of the maximum possible score (0–100%)

*Abbreviations*: *No* number of questions, *Min* Minimum, *Max* Maximum

An analysis of the pre-test performance in relation to the covariates gender, course affiliation, and age revealed a significant effect of course affiliation on performance *(p* = 0.013) (Table 3).

**Table 3:** Influence of covariates on pre-test performance

Category		*p*-value^*^
Gender, n (%), mean Pre-test (%) ± SD)		0.598^†^
Male	24 (30.38),89.90 ± 9.34	
Female	55 (69.62),89.27 ± 7.69	
Total	79 (100),89.46 ± 8.17	
Course Affiliation, n (%), mean Pre-test (%) ± SD		0.013^‡^
IC1	16 (20.3), 86.25 ± 8.99	
IC2	14 (17.7), 87.14 ± 6.27	
IC3	32 (40.5), 89.61 ± 7.78	
IC4	17 (21.5), 94.12 ± 7.90	
Total	79 (100), 89.46 ± 8.17	
Age (years), n (%), mean Pre-test (%) ± SD		0.784^§^
21	2 (2.5), 90.00 ± 14.14	
22	8 (10.13), 90.31 ± 8.49	
23	11 (13.92), 86.14 ± 8.61	
24	4 (5.06), 84.38 ± 3.14	
25	10 (12.66), 92.25 ± 9.01	
26	8 (10.13), 90.00 ± 7.67	
27	8 (10.13), 91.88 ± 8.10	
28	7 (8.86), 90.00 ± 8.78	
29	5 (6.33), 87.00 ± 11.09	
30	4 (5.06), 91.25 ± 7.21	
31	2 (2.5), 96.25 ± 5.30	
32	3 (3.80), 85.00 ± 10.00	
33	1 (1.27), 90.00 ± —	
34	1 (1.27), 85.00 ± —	
36	1 (1.27), 82.50 ± —	
38	1 (1.27), 95.00 ± —	
40	1 (1.27), 87.50 ± —	
43	1 (1.27), 100.0 ± —	
51	1 (1.27), 85.00 ± —	

*Abbreviations*: *IC* interdisciplinary course (IC 1–4 correspond to semesters 7–10, respectively), *n* number of participants

^*^Statistical significance level *p* < 0.05

^**†**^Mann Whitney U

^‡^Kruskal Wallis

^§^linear Regression

After applying the Benjamini–Hochberg correction to the pairwise course comparisons, only the pre-test difference between IC2 and IC4 remained significant (adjusted p = 0.023). The pre-test differences between IC1 and IC4 and between IC3 and IC4 were not significant (adjusted p = 0.060 for both) (Table 4).

**Table 4:** Pairwise results for course group comparisons in pre-test performance

Course affiliation	Adjusted *p*-Value^* †^
IC1 vs. IC2	0.629
IC1 vs. IC3	0.140
IC1 vs. IC4	0.060
IC2 vs. IC3	0.202
IC2 vs. IC4	**0.023**
IC3 vs. IC4	0.060

*Abbreviations*: *IC* interdisciplinary course (IC 1–4 correspond to semesters 7–10, respectively)

^*^Statistical significance level *p* < 0.05

^†^After Benjamini–Hochberg correction

### Post-test

Results after the case assessment are outlined in Table 5.

**Table 5:** Post-test results

Category	Root canal number	Vertucci-classification	Complexity
Mandibular anterior tooth			
2D^**†**^ (%)	83.30	97.60	90.50
3D^**‡**^ (%)	97.30	94.60	91.90
Difference 2D/3D (%)	−14.00	3.00	−1.32
OR 3D/2D	7.20	0.43	1.19
95% CI	0.84–61.59	0.04–4.91	0.25–5.72
Adjusted *p*-value^*^	0.099	0.556	0.874
Maxillary anterior tooth			
2D^**†**^ (%)	97.20	70.30	64.90
3D^**‡**^ (%)	92.90	92.90	97.60
Difference 2D/3D (%)	4.30	−22.60	−32.70
OR 3D/2D	0.37	5.50	22.21
95% CI	0.04–3.74	1.40–21.64	2.73–180.53
Adjusted *p*-value^*^	0.481	0.044^*^	0.013^*^
Mandibular premolar			
2D^**†**^ (%)	94.60	89.20	97.30
3D^**‡**^ (%)	95.20	78.60	73.80
Difference 2D/3D (%)	−0.60	10.60	23.50
OR 3D/2D	1.14	0.44	0.08
95% CI	0.15–8.55	0.12–1.59	0.01–0.64
Adjusted *p*-value^*^	0.897	0.272	0.045^*^
Maxillary premolar			
2D^**†**^ (%)	52.40	14.30	45.20
3D^**‡**^ (%)	97.30	32.40	70.30
Difference 2D/3D (%)	−44.90	−18.10	−25.10
OR 3D/2D	32.73	2.88	2.86
95% CI	4.10–261,25	0.95–8.69	1.13–7.26
Adjusted *p*-value^*^	0.006^**^	0.091	0.054
Mandibular molar			
2D^**†**^ (%)	45.20	52.40	59.50
3D^**‡**^ (%)	81.10	27.00	21.60
Difference 2D/3D (%)	−35.90	25.40	37.90
OR 3D/2D	5.19	0.34	0.19
95% CI	1.87–14.43	0.13–0.87	0.07–0.51
Adjusted *p*-value^*^	0.007^**^	0.054	0.006^**^
Maxillary molar			
2D^**†**^ (%)	35.10	35.10	67.60
3D^**‡**^ (%)	92.90	59.50	45.20
Difference 2D/3D (%)	−57.80	−24.40	22.40
OR 3D/2D	24	2.72	0.40
95% CI	6.19–92.99	1.09–6.77	0.16–0.99
Adjusted *p*-value^*^	< 0.001^***^	0.058	0.079

The model-derived estimated marginal probabilities, odds ratios, and adjusted *p*-values depend on the image presentation for classification accuracy. The percentages represent the estimated marginal probabilities from the logistic mixed-effects models. Odds ratios compare holography with periapical radiography (3D versus 2D). The *p*-values were adjusted for multiple testing across 18 case- and outcome-specific contrasts using the Benjamini–Hochberg false discovery rate procedure. An OR greater than 1 favors holography, while an OR less than 1 favors periapical radiography

*OR* Odds ratio, *CI* confidence interval

^*^Statistical significance level *p* < 0.05

^**^p < 0.01

^***^p < 0.001

^†^Periapical radiograph

^‡^Holography

After the Benjamini–Hochberg correction, holograms significantly improved the identification of the number of root canals in three cases: the maxillary molar (Case 1: = OR 24, 95% CI 6.19–92.99, adjusted p = 0.0001), the maxillary premolar (Case 5: = OR 32.73, 95% CI 4.1–261.25, adjusted p = 0.006) and the mandibular molar (Case 6: OR = 5.19, 95% CI 1.87–14.43, adjusted p = 0.007). In the other cases, no significant improvement could be observed after correction.

For Vertucci classification, only the maxillary anterior tooth remained significant after correction (Case 3: OR = 5.5, 95% CI 1.40–21.64, adjusted p = 0.044). The nominally significant maxillary molar and mandibular molar did not survive multiplicity correction and are therefore interpreted cautiously.

After correction, the effect of treatment complexity was case-dependent. Radiography was superior for the mandibular premolar (Case 2: OR = 0.08, 95% CI 0.01–0.64, adjusted p = 0.045) and mandibular molar (Case 6: OR = 0.19, 95% CI 0.07–0.51, adjusted *p* = 0.006), while holography was superior for the maxillary anterior tooth (Case 3: OR = 22.21, 95% CI 2.73–180.53, adjusted *p* = 0.013). The differences for the maxillary molar and premolar were not significant after multiplicity correction and should be interpreted cautiously.

**Fig. 6:**
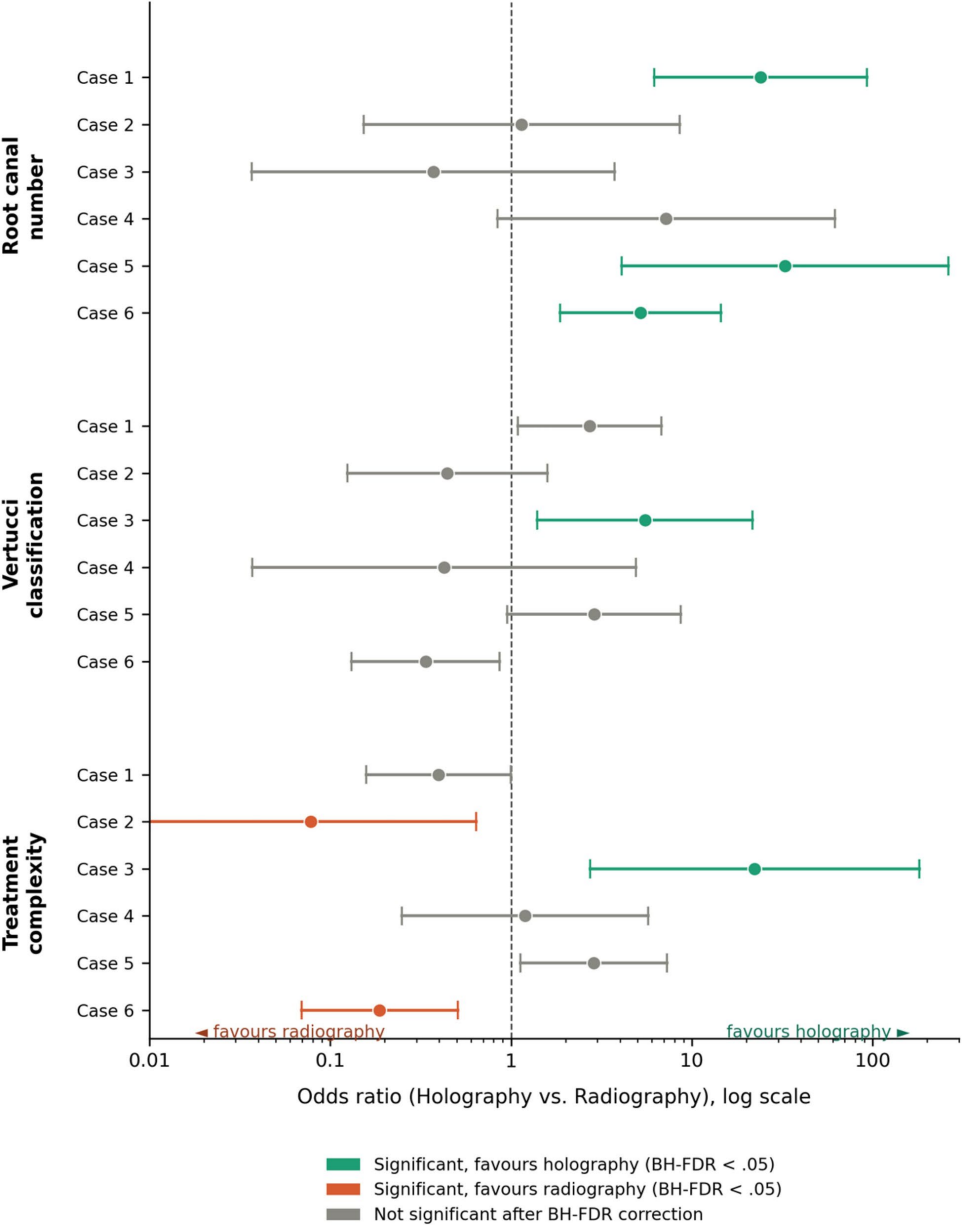
Per-case treatment effects with 95% confidence intervals. Odds ratios and 95% confidence intervals for case-specific comparisons of holography versus periapical radiography. *P*-values were adjusted across all 18 contrasts using the Benjamini–Hochberg false-discovery-rate procedure. Odds ratios greater than 1 favour holography; odds ratios below 1 favour periapical radiography

Figure 6 shows the odds ratios and 95% confidence intervals for all 18 contrasts. The Benjamini–Hochberg false-discovery-rate procedure was used to adjust *p*-values across all contrasts.

### Evaluation questionnaire

Higher participant satisfaction was achieved with hologram technology compared to conventional periapical radiographs across all diagnostic categories: 82.3% rated it superior for canal count determination, 59.5% for Vertucci classification, and 43.0% for assessing treatment complexity (percentages correspond to the best rating on the 5-point Likert scale). Regarding user comfort, 77.2% reported no physical discomfort during or after using the holographic interface. Most participants (97.5%) supported the integration of hologram technology into future dental education, with 54.4% advocating for its immediate adoption in clinical training (Table 6).

**Table 6:** Evaluation questionnaire

No	Total (N)	Question	Response Format	n (%)	mean ± SD	Minimum	Maximum
1	79	Annotation of root canal number (Holography vs. periapical radiograph)	5-point comparative Likert scale				
			1. significantly better	65 (82.28)	—	—	—
			2.better	13 (16.45)	—	—	—
			3. equal	1 (1.27)	—	—	—
			4. worse	—	—	—	—
			5. significantly worse	—	—	—	—
2	78	Annotation of Vertucci classification (Holography vs. periapical radiograph)	5-point comparative Likert scale				
			1. significantly better	47 (60.26)	—	—	—
			2.better	27 (34.62)	—	—	—
			3. equal	3 (3.85)	—	—	—
			4. worse	1 (1.28)	—	—	—
			5. significantly worse	—	—	—	—
3	79	Assessment of treatment complexity (Holography vs. periapical radiograph)	5-point comparative Likert scale				
			1. significantly better	34 (43.04)	—	—	—
			2.better	34 (43.04)	—	—	—
			3. equal	11 (13.92)	—	—	—
			4. worse	—	—	—	—
			5. significantly worse	—	—	—	—
4	79	Innovative potential of holographic visualization	VAS (0–100) 0 = not innovative 100 = very innovative	—	83.68 ± 19.68	1.000	100.0
5	79	Innovative potential of holographic visualization in other dental disciplines	VAS (0–100) 0 = not innovative 100 = very innovative	—	64.64 ± 23.81	0.000	100.0
6	69	Dental discipline with the greatest potential for holographic visualization	Single-choice question				
			1. Tooth preservation & periodontology	52 (75.36)	—	—	—
			2. Dental prosthetic	2 (2.90)	—	—	—
			3. Orthodontics	4 (5.80)	—	—	—
			4. Oral surgery	11 (15.94)	—	—	—
			5. Pediatric dentistry	—	—	—	—
7	79	Innovative potential of holographic visualization in patient education	VAS (0–100) 0 = not innovative 100 = very innovative	—	82.72 ± 20.72	20.00	100.0
8	79	Good usability of holographic visualization	VAS (0–100) 0 = do not agree 100 = fully agree	—	83.33 ± 16.02	30.00	100.0
9	78	Evaluation of the hologram size	5-point comparative Likert scale				
			1. significantly undersized	1 (1.28)	—	—	—
			2. undersized	5 (6.41)	—	—	—
			3. excellent	42 (53.85)	—	—	—
			4. oversized	27 (34.62)	—	—	—
			5. significantly oversized	3 (3.85)	—	—	—
10	78	Evaluation of the animation duration	5-point comparative Likert scale				
			1. significantly too short	—	—	—	—
			2. too short	22 (28.21)	—	—	—
			3. excellent	37 (47.44)	—	—	—
			4. too long	19 (24.36)	—	—	—
			5. significantly too long	—	—	—	—
11	79	Technical implementation of the holographic visualization	VAS (0–100) 0 = very bad 100 = excellent	—	75.50 ± 16.99	40.00	100.0
12	78	Physical symptoms with holographic visualization	Single-choice question				
			1. Dizziness	3 (3.85)	—	—	—
			2. Headache	1 (1.28)	—	—	—
			3. Eye pain	4 (5.13)	—	—	—
			4. Tiredness	3 (3.85)	—	—	—
			5. Blurred vision	6 (7.69)	—	—	—
			6. None	61 (78.21)	—	—	—
13	79	Integration of holographic visualization in dental education	Binary question				
			1. Yes	77 (97.47)	—	—	—
			2. No	2 (2.53)	—	—	—
13b	77	If the answer to question 13 is yes, from which semester?	Single-choice question				
			1. Clinical simulation course	43 (55.84)	—	—	—
			2. Interdisciplinary Course 1	27 (35.06)	—	—	—
			3. Interdisciplinary Course 2	1 (1.30)	—	—	—
			4. Interdisciplinary Course 3	4 (5.19)	—	—	—
			5. Interdisciplinary Course 4	2 (2.60)	—	—	—

Assessment items to evaluate the holography system

*Abbreviations*: *No* number of question, *N* total number of participants (due to incorrectly completed evaluation forms, the total number varies for some questions), *n* number of participants, *VAS* visual analog scale

## Discussion

To evaluate the use of hologram technology by students in endodontic diagnostics, we compared the precision of endodontic diagnoses using hologram animations with those using conventional radiographs. The findings suggest that the benefits of holographic visualization are task dependent. Holograms significantly improved the identification of number of root canals in multi-rooted teeth and improved the assessment of Vertucci classification in one case significantly. However, in certain cases conventional radiography remained significantly superior for the evaluation of treatment complexity. Despite these mixed objective findings, participants perceived hologram technology as highly innovative and strongly supported its integration into future undergraduate dental curricula.

The utilization of hologram visualization in the determination of the number of root canals in multi-rooted teeth has been demonstrated to enhance the accuracy of the procedure. In contrast, no discernible difference was observed in single-rooted teeth, irrespective of the form of visualization employed. In two-dimensional visualization methods, the risk of overlapping of certain structures and the resulting risk of overlooked canals is increased in multi-rooted teeth due to the axial position of individual roots [31]. The utilization of three-dimensional hologram animation facilitates the circumvention of these overlaps, thereby conferring a discernible added value in the visualization of complex and multi-rooted teeth. Our findings are also relevant when compared to other studies, as those studies have already shown that students describe the interpretation of radiographs and the treatment of molars in endodontics as particularly challenging. Tavares et al. (2019) identified the radiographic interpretation of the number of root canals as particularly challenging for students, while Davey et al. (2015) describe lower confidence in treating multi-rooted molars compared to single-rooted teeth [5, 32]. Holographic visualization could be a promising approach here to assist in the radiographic interpretation of the number of root canals in multi-rooted teeth.

In determining the Vertucci classification, the hologram demonstrated higher accuracy for maxillary anterior teeth. The Vertucci classification is not incorporated into the endodontic training curriculum of dental studies and was first presented in the calibration seminar. Consequently, the allocation of cases to the eight subtypes of the classification may prove to be overly onerous. The inconsistent results of the hologram in determining channel configuration could thus be explained by the fact that correct classification involves not only anatomical visualization but also the transfer of the visualized information to an abstract classification system. The results should also be further examined to determine whether the inherent complexity of the Vertucci classification may have influenced the accuracy of the classification using holographic visualization. The classification requires the identification of individual canal structures, the determination of their three-dimensional courses, and their assignment to one of eight defined types [23]. Ahmed et al. (2017) describe these predefined type classifications as having limited applicability due to the marked variability of the root canal system and the severely limited ability to visualize this complex anatomy [33]. The challenging complexity of the classification, combined with the theory taught only during the calibration seminar, may have reduced the effectiveness of holographic visualization in this regard.

In the assessment of mandibular premolar and molar complexity, we found that hologram imaging was inferior to conventional radiographs. The treatment complexity of AAE represents a complex, multifactorial construct that goes far beyond mere anatomical visualization. Even when considering radiographic criteria alone, classifying them into the corresponding levels of complexity remains challenging. According to cognitive load theory, the simultaneous interpretation of the visualized anatomy and the integration of multiple pieces of information constitute a mentally demanding task [34]. Accordingly, this cognitive load may have overburdened the students’ cognitive capacities and thus diminished the educational effectiveness of the holograms. The novelty of the technology may have overwhelmed the students, particularly when it came to solving complex tasks such as assessing complexity [34].

Despite this mixed objective classification accuracy of holographic visualization, students find it very innovative and express a clear desire to see it incorporated into future dental curricula. Previous studies on virtual and augmented reality applications in dental education report similar findings regarding students’ subjective perceptions of immersive and digital methods [35]. In a comparative study by Reymus et al. (2020) on the evaluation of VR technology for the visualization of root canal anatomy, significantly better results were shown for case assessment in the VR environment compared to analysis using conventional periapical radiographs [8].

Research has identified positive effects of digital technologies on teaching in other areas of dentistry. For instance, Liebermann et al. (2024) presented prosthetic patient cases in a VR environment to both students and teachers at various educational institutions [25]. Their findings indicated a high level of satisfaction among the participants with this learning method and a clear recommendation for its further integration into dental training.

The application of digital technologies in education is also being evaluated in fields beyond dentistry. Bork et al. (2019) conducted a comparative analysis of anatomical knowledge before and after the implementation of classic anatomy atlases, virtual dissection tables, and a learning system based on AR [36]. In addition to the high level of satisfaction expressed by participants with the digital learning methods, a significant improvement in anatomical knowledge was observed among subjects who used the AR system.

Based on our results, we can only partially confirm our hypothesis that holographic visualizations improve both the objective classification accuracy and students’ subjective perception of teaching effectiveness compared to conventional periapical radiographs. Rather than demonstrating general superiority across all classification parameters examined, the holographic visualization shows only task-specific superiority, particularly in determining the number of canals in multi-rooted teeth. In terms of subjective evaluation, the hologram meets our expectations as an innovative and appealing visualization tool.

To the best of our knowledge our study is distinctive in that it was the first to visualize free-standing holograms using a three-dimensional projector without the need for supplementary equipment, such as specialized glasses or headsets. While other studies have primarily used virtual and augmented reality to visualize anatomical structures or to assess the subjective perception of immersive technologies, our study visualized real patient cases and determined the accuracy of diagnostic tasks, such as determining the number of root canals, canal configuration, and treatment complexity, when using holographic technology. Furthermore, both objective classification accuracy and subjective assessment were measured, allowing for a discussion of both the diagnostic and didactic potential of holograms. Our study therefore offers new insights into the benefits of using holographic visualizations and provides a foundation for further research into future teaching concepts.

Despite these strengths, several limitations should be considered when interpreting the present findings.

We conducted a pre-test to document the prior knowledge of the participants before the start of the study, which may have differed between IC. A subsequent analysis of the pre-test results revealed a significant influence of course affiliation on the percentage achieved. Participants in their final year of dental school (IC 4) showed a higher percentage than all other courses (IC 1–3). The better results can be attributed to the extended practical experience of students in IC 4 in treating their own patients and to the more extensive theoretical knowledge they had acquired during their studies. The high pre-test score already suggests excellent prior theoretical knowledge regarding root canal anatomy and the AAE complexity classes. However, the pre-test did not assess spatial understanding or the ability to interpret three-dimensional anatomical visualizations. For this reason, we assume that the pre-test had no influence on the determination of the number of root canals or the identification of the corresponding structures. Nevertheless, the already high pre-test scores may have limited the hologram’s potential advantage over conventional periapical radiographs in assigning visualized structures to existing classification systems, such as those developed by Vertucci or the AAE.

As has been previously demonstrated in studies on VR and AR applications, the phenomenon of cybersickness is a potential limitation [37]. The presence of symptoms such as fatigue, headaches, and dizziness has been observed in association with the use of VR technologies [38], which can manifest during or following the utilization of these technologies. In the period preceding our study, the occurrence of such symptoms when observing holograms could not be entirely discounted. However, approximately 77% of participants did not exhibit any symptoms of cybersickness. Although most of our participants were free of symptoms of cybersickness, future studies require larger cohorts and appropriate measures of cybersickness to draw definite conclusions about the safety of using holograms. To minimize potential health risks, students with preexisting conditions such as epilepsy, migraines, or vertigo were excluded from the study. Therefore, our results may not apply to all student populations. Further studies regarding the safety of holographic visualizations are needed before these groups can be included.

A further constraint of our study is the small number of cases. A total of six cases in all tooth groups were presented to each participant in both presentation methods. Consequently, while the study does illustrate variations in endodontic anatomy contingent on tooth groups, it does not address deviations within individual tooth groups. Since each tooth group was represented by only one case, it is difficult to distinguish modality effects from case-specific characteristics. Therefore, the results should be interpreted exploratory and not generalized to entire tooth groups. To achieve a more comprehensive depiction of endodontic holography, it is imperative that future studies encompass a greater number of cases. Since the analysis included only 79 of the planned 90 students, the study may be limited in its ability to detect individual differences in case-specific comparisons.

Although the visualization modality was randomized using a crossover design, the six cases were presented to all students in the exact same order. Consequently, learning effects cannot be completely ruled out, as the participants became increasingly familiar with the Vertucci classification and the criteria for treatment complexity. These learning effects are more likely to affect the knowledge-based tasks of configuration determination and complexity assessment than the determination of the number of root canals.

Given our total exposure time of 18 min, the risk of fatigue is rather low. Studies typically report signs of fatigue and a decline in performance among participants for tasks lasting at least 20 min [39, 40]. While minor signs of fatigue cannot be ruled out, they play a minor role in the interpretation of the results.

This study also examined the assessment of treatment complexity based on the AAE’s radiological criteria. Although these criteria generally allow for the classification of treatment complexity, the results should be viewed in the context of an imaging-based assessment and not as a substitute for a comprehensive clinical case evaluation.

In addition, the results of the evaluation questionnaire should be interpreted with caution. Since no validated questionnaires exist for this area of application, an exploratory questionnaire was designed. Furthermore, the students were shown a new type of technology, which can lead to novelty bias, social desirability bias, and expectation effects. Consequently, the predominantly positive subjective perception should not be equated with the objective teaching effectiveness of holographic projection, since the hologram is inferior to periapical radiograph, particularly when assessing treatment complexity. One possible explanation for this is that, due to its immersive and innovative nature, holographic projection may have increased engagement and the perceived learning effect without influencing diagnostic performance in determining root canal configuration and assessing complexity [41].

The temporal context of the case assessment may have also exerted an influence on the outcomes. The execution of this endeavor could only be accomplished in a concomitant manner with the participants’ prevailing teaching program, entailing multiple sessions and groups of approximately 12 participants. Due to this difficulty in conducting the study, the time interval between the calibration seminar and case assessment ranges from two to six weeks. The temporal discrepancy between the presentation of the case and the preceding calibration seminar may have exerted an influence on the results obtained by the participants.

In addition to these educational limitations, there are also several technical limitations associated with holographic visualization that should not be overlooked. Holograms were created from CBCT scans, depicting only the crown and root structures, including the root canal system. Other factors essential for endodontic treatment planning, such as the condition of the periapical tissue, tooth position, and tooth angulation, could not be visualized. Furthermore, fine anatomical details may have been lost during the segmentation of the teeth using 3D Slicer and the post-processing in Blender. The complex production process for the holograms also currently prevents their rapid incorporation into dental education.

Because of the study design, it was not possible to blind the subjects or investigators. This should be considered when evaluating the results.

## Conclusions

Holographic visualization had case- and task-specific effects on endodontic training. Compared to conventional periapical radiographs, holograms were associated with higher classification accuracy in determining the number of root canals in multi-rooted teeth. The superiority of holography was also demonstrated in one case when determining the configuration according to Vertucci, whereas conventional dental films appear to be superior in certain cases to holograms in assessing treatment complexity. Despite the predominantly positive subjective perception of hologram technology, it should not be equated with general superiority as a teaching method. Holographic visualizations could offer advantages as a supplementary method in certain endodontic cases. However, further studies are needed to assess their impact on broader educational and clinical outcomes.

## Supplementary Information

Supplementary Material 1.

## Data Availability

The datasets used and/or analysed during the current study are available from the corresponding author on reasonable request.

## Abbreviations

CBCT: Cone-beam computed tomography
VR: Virtual Reality
AR: Augmented Reality
IC: Interdisciplinary Course
AAE: American Association of Endodontists
3D: Three-dimensional
STL: Standard Tesselation Language
cm: Centimeter
VAS: Visual Analog Scale
CI: Confidence interval
BOBYQA: Bound Optimization BY Quadratic Approximation
OR: Odds ratio
